# World Single Age Records in Running From 5 km to Marathon

**DOI:** 10.3389/fpsyg.2018.02013

**Published:** 2018-10-23

**Authors:** Beat Knechtle, Pantelis T. Nikolaidis, Stefania Di Gangi

**Affiliations:** ^1^Medbase St. Gallen Am Vadianplatz, St. Gallen, Switzerland; ^2^Institute of Primary Care, University of Zurich, Zurich, Switzerland; ^3^Exercise Physiology Laboratory, Nikaia, Greece

**Keywords:** female, male, aging, youth, performance

## Abstract

This study investigated the relationship between race times and age, in 1-year intervals, by using the world single age records, from 5 km to marathon running (i. e., 5 km, 4 miles, 8, 10, 12, 15 km, 10 miles, 20 km, half-marathon, 25 km, 30 km, and marathon). For each race, a regression model was fitted. Effects of sex, alone and in interaction with age, and the effect of country of origin on performance were examined in a multi-variable model. The relationship between age and race time was modeled through a 4th order-polynomial function. Women achieved their best half-marathon and marathon race time, respectively, 1 year and 3 years earlier in life than men. On the contrary, in the other races, the best women performances were achieved later in life than men (i.e., 4 miles and 30 km: 2 years later, 8 km: 3 years later, 15–20–25 km: 1 year later, 10 miles: 4 years) or at the same age (i.e., 5, 10, 12 km). Moreover, age of peak performance did not change monotonically with the distance of race. For all races, except 12 km, sex differences had an absolute maximum at old ages and a relative maximum near the age of peak performance. From 8 km onward, estimated sex differences were increasing with increasing race distance. Regarding country, runners from Canada were slower than runners from the United States of America in 5 km by 00:10:05 h:min:s (*p* < 0.001) and in half-marathon by 00:18:43 h:min:s (*p* < 0.01). On the contrary, in marathon, they were 00:18:43 h:min faster (*p* < 0.05). Moreover, in 10 miles, runners from Great Britain were 00:02:53 h:min:s faster (*p* < 0.05) than runners from the United States of America. In summary, differences seem to exist in the age of peak performance between women and men and for nearly all distances sex differences showed an absolute maximum at old ages and relative maximum near the age of peak performance. Thus, these findings highlight the need for sex-specific training programs, especially near the age of peak performance and for elder runners.

## Introduction

It is well known that for each sports discipline a specific age of peak athletic performance exists (Allen and Hopkins, 2015). Generally, this age of peak performance increases with increasing duration of an endurance performance (Allen and Hopkins, 2015). This is especially obvious in ultra-marathon running, where the age of peak athletic performance increases with increasing race duration when ultra-marathons from 6 h to 10 days were investigated (Knechtle et al., 2014). Accordingly, when two of the most popular ultra-marathon distances, the 50 km (Nikolaidis and Knechtle, 2018a) and the 100 km (Nikolaidis and Knechtle, 2018b), were analyzed, the age of peak running performance was older in the longer running distance.

For running, the age of peak athletic performance has mainly been investigated for half-marathon (Knechtle and Nikolaidis, 2018; Nikolaidis et al., 2018), marathon (Lara et al., 2014; Knechtle et al., 2017; Nikolaidis and Knechtle, 2018b,c; Nikolaidis et al., 2018), and ultra-marathon (Knechtle and Nikolaidis, 2017) running. However, the age of peak athletic performance in shorter endurance running distances has not been studied previously to the best of our knowledge.

It is also important for athletes and coaches to be aware of potential differences in the age of peak athletic performance between the sexes. Regarding a systematic review investigating the age of peak athletic performance for different events (i.e., explosive power/sprint, endurance, mixed/skill), it was showed that there was little difference in estimates of peak age for these event types between men and women (Allen and Hopkins, 2015). Nevertheless, very recent studies showed differences in the age of peak athletic performance between women and men. For example, in half-marathon (Knechtle and Nikolaidis, 2018; Nikolaidis and Knechtle, 2018a; Nikolaidis et al., 2018) and in marathon running (Nikolaidis and Knechtle, 2018b; Nikolaidis et al., 2018), women achieved their best race times earlier in life than men in studies where all runners by age group were considered. These findings were in contrast to earlier findings where the age of peak marathon performance was assumed to appear earlier in life in men compared to women in a research where top 10 runners by age group were considered (Lara et al., 2014). When world class track-and-field athletes were investigated, the mean age of peak performance was older for marathon and male throwers whereas women reached an older age of peak performance than men in the hurdles, middle and long-distance running events. Furthermore, male throwers had a higher peak age than female throwers (Haugen et al., 2018).

A confounding variable that might influence the age of peak performance is nationality. The prevalence of particular nationalities in the endurance running has been documented recently (Nikolaidis et al., 2017a,b). For instance, half of participants in all running events of the World Championships from 1975 to 2015 were from USA, Germany, Australia, and Great Britain (Nikolaidis et al., 2017b), whereas most runners in the 10 km event, half-marathon and marathon were Kenyans according to the data of the International Association of Athletics Federations 1999–2015 (Nikolaidis et al., 2017a). In addition, differences in age of runners among nationalities were observed (Nikolaidis et al., 2017a). Thus, it would be reasonable to assume that nationality should be considered when analyzing the relationship between age and performance.

To gain more insight in the age of peak running performance for shorter running distances and the role of nationality, this study aimed at analyzing the world single age records in road races from 5 km to marathon for the distances of 5 km, 4 miles, 8 km, 10 km, 12 km, 15 km, 10 miles, 20 km, half-marathon, 25, 30 km, and marathon by using data of the of the “Association of Road Racing Statisticians” (ARRS). Based upon the findings that the age of peak athletic performance gets older while the race distance becomes longer in ultra-marathon running, the research hypothesis was to expect a younger age of peak performance in the shorter compared to the longer distances of road running races.

## Materials and methods

### Ethical approval

The institutional review board of St Gallen, Switzerland, approved this study. Since the study involved analysis of publicly available data, the requirement for informed consent was waived.

### Data sampling and data analysis

The data set for this study was obtained from the website of the ARRS (https://arrs.run/SARec.htm) from 5 km to marathon for both women and men. The distances 5 km, 4 miles, 8, 10, 12, 15 km, 10 miles, 20 km, half-marathon, 25 km, 30 km, and marathon were considered. It should be highlighted that all distances referred to road running races. This section lists the fastest performances for each single age and for each of the standard distances, as known to ADR. Performances are subject to the same standards as listing for national records plus the additional requirement that the runner's date of birth (as well as the race date) must be known. These are required to be able to document the runner's exact age at the time of the performance. Single age records that meet the ARRS qualifying standards may be expected to be fairly reliable. At older (and younger) ages, the best marks known to ARRS are listed. We used the full data set without data cleaning.

#### Statistical analyses

All data have been presented as means and standard deviations. Time was recorded in the format “h:m:s.” For each race, *t*-tests were performed to compare the average performance between genders by age-groups and by country. Age-groups were [3,20), [20,30), [30,40), [40,50), [50,60), [60,75), [75,100). The most prevalent countries, in terms of participation, were USA, Canada (CAN), Kenya-Ethiopia (KEN-ETH) and, in 30 km and marathon also Japan (JPN). The other countries were grouped together. Age was considered as a continuous variable, in 1 year intervals, when defined as a predictor variable for race-time. A non-linear regression mixed model, with a fourth order (i.e., quartic) polynomial function, was performed to find the age of peak performance, which was the age at which the time record-fitted value had a minimum. The mixed model was used to correct for repeated measurements within runners (clusters) through the random effects of intercepts. Gender, country and age-gender interaction term, were also considered as predictors, fixed effects. In other words, for each race, from 5 km to marathon, the statistical model was specified as follows:

Race Time (Y) ~ (Fixed effects (X) = Sex + (P_Age_+${\text{P}}_{\text{Age}}^{2}$ + ${\text{P}}_{\text{Age}}^{3}+$ ${\text{P}}_{\text{Age}}^{4}$) + Sex × (P_Age_ + ${\text{P}}_{\text{Age}}^{2}$ + ${\text{P}}_{\text{Age}}^{3}$ + ${\text{P}}_{\text{Age}}^{4}$) + Country] + (Random effects of intercept = Runners]

where × denoted the interaction function and (P_Age_ + ${\text{P}}_{\text{Age}}^{2}$ + ${\text{P}}_{\text{Age}}^{3}$ + ${\text{P}}_{\text{Age}}^{4}$) a polynomial function on age of grade 4.

As a measure of goodness of fit of each model, the r-squared approximation (*R*^2^) and a simplified version of the Omega-squared value ($\Omega_{0}^{2}$) were computed. *R*^2^ is the correlation between the fitted and observed values and $\Omega_{0}^{2}$ is defined as 1–(residual variance/response variance). Moreover, the Intraclass Correlation Coefficient (ICC), which is a measure of how strongly units in the same cluster resemble each other, has been reported for each model.

In addition, sex differences in performance were examined and were defined, in percentages, as 100 × (women's race time-men's race time)/men's race time. For all tests and regressions, statistical significance was defined at *p* < 0.05. All statistical analyses were carried out using statistical package R, R Core Team (2016). R: A language and environment for statistical computing. R Foundation for Statistical Computing, Vienna, Austria. URL https://www.R-project.org/. Packages ggplot2, lme4, and lmerTest were used, respectively, for data visualization and for the mixed model.

## Results

### Summary statistics

In Table 1, for each race from 5 km to marathon, the numbers of observations and the average performances by sex, age-groups, and countries have been reported. For each race, there was no significant difference between sex performances in the first age group [3, 20). For ages from 20 (included) to 60 excluded, differences between sex performances were significant (*p* < 0.001 for quite all cases) in all races. For ages [60, 75), differences were significant for all races excluded 20 km. In the last age group [75, 100), differences were significant between sex performances only in 5 km (*p* = 0.039), 10 mi (*p* = 0.028), and marathon (*p* = 0.010).

**Table 1:** Mean performance of world records (5 km to marathon) by sex, age-groups, and country.

		**5 km**, ***N*** = **183**				**4 mi**, ***N*** = **151**				**8 km**, ***N*** = **167**				**10 km**, ***N*** = **171**			
	**Sex**	***N***	**Mean**	**Sd**	***p***	***N***	**Mean**	**Sd**	***p***	***N***	**Mean**	**Sd**	***p***	***N***	**Mean**	**Sd**	***p***
**AGE**																	
[3,20)	Men	17	00:18:49	00:06:45	0.702	13	00:25:36	00:09:28	0.959	14	00:29:56	00:08:03	0.890	13	00:34:33	00:06:43	0.385
[3,20)	Women	17	00:19:46	00:07:38		11	00:25:45	00:04:44		14	00:29:37	00:03:41		14	00:36:41	00:05:45	
[20,30)	Men	10	00:13:14	00:00:04	<0.001	10	00:17:36	00:00:06	<0.001	10	00:22:09	00:00:07	<0.001	11	00:27:15	00:00:14	<0.001
[20,30)	Women	10	00:14:55	00:00:07		11	00:20:09	00:00:31		10	00:25:02	00:00:20		10	00:30:27	00:00:21	
[30,40)	Men	10	00:13:32	00:00:09	<0.001	10	00:18:09	00:00:27	<0.001	11	00:22:33	00:00:18	<0.001	10	00:27:38	00:00:21	<0.001
[30,40)	Women	10	00:15:14	00:00:18		10	00:20:07	00:00:14		11	00:25:22	00:00:39		10	00:31:09	00:00:24	
[40,50)	Men	10	00:14:22	00:00:30	<0.001	10	00:19:25	00:00:49	<0.001	10	00:23:55	00:00:41	<0.001	10	00:29:31	00:01:07	<0.001
[40,50)	Women	10	00:16:11	00:00:17		10	00:22:20	00:01:01		10	00:26:36	00:00:42		11	00:33:15	00:00:41	
[50,60)	Men	10	00:15:44	00:00:38	<0.001	10	00:21:43	00:00:37	<0.001	10	00:26:01	00:00:54	<0.001	10	00:32:11	00:00:52	<0.001
[50,60)	Women	10	00:17:52	00:00:49		10	00:25:05	00:01:42		10	00:29:49	00:01:30		11	00:36:10	00:01:24	
[60,75)	Men	15	00:17:38	00:01:06	<0.001	14	00:24:09	00:01:29	<0.001	15	00:29:50	00:02:22	<0.001	15	00:36:12	00:02:00	<0.001
[60,75)	Women	15	00:21:00	00:01:50		12	00:31:40	00:03:16		16	00:35:28	00:02:50		15	00:42:48	00:03:20	
[75,100)	Men	21	00:29:37	00:08:37	0.039	14	00:43:26	00:14:36	0.681	16	00:49:16	00:22:21	0.948	18	00:55:50	00:15:33	0.405
[75,100)	Women	18	00:42:57	00:24:21		6	00:41:15	00:08:27		10	00:48:52	00:07:52		13	01:00:29	00:14:46	
**COUNTRY**																	
USA	Men	36	00:23:30	00:10:24	0.443	41	00:29:45	00:13:33	0.392	30	00:38:01	00:19:58	0.108	12	00:38:57	00:08:44	0.431
USA	Women	46	00:25:51	00:16:52		48	00:27:42	00:07:23		31	00:31:45	00:05:49		13	00:43:41	00:19:09	
CAN	Men	13	00:19:25	00:02:18	0.151	5	00:22:51	00:01:44		13	00:33:11	00:06:52	0.009	11	00:40:50	00:04:17	0.142
CAN	Women	4	00:48:53	00:30:44		1	00:23:37			10	00:45:24	00:11:10		2	01:07:38	00:09:19	
ETH-KEN	Men	18	00:13:18	00:00:14	<0.001	17	00:18:00	00:00:32	<0.001	17	00:22:21	00:00:27	<0.001	25	00:27:36	00:00:31	<0.001
ETH-KEN	Women	14	00:15:05	00:00:24		10	00:20:13	00:00:22		10	00:25:28	00:00:31		11	00:30:43	00:00:34	
GBR	Men	21	00:17:18	00:03:37	0.062	12	00:24:44	00:07:59	0.287	16	00:27:17	00:05:06	0.011	16	00:38:03	00:15:10	0.742
GBR	Women	12	00:19:43	00:03:18		3	00:29:24	00:05:24		13	00:32:39	00:05:23		19	00:39:25	00:06:49	
JPN	Men																
JPN	Women																
Other	Men	5	00:14:43	00:01:01	0.021	6	00:18:34	00:01:25	0.018	10	00:23:34	00:01:18	<0.001	23	00:42:24	00:16:58	0.422
Other	Women	14	00:16:42	00:02:22		8	00:20:31	00:00:55		17	00:26:17	00:01:30		39	00:39:16	00:09:20	
		**12 km**, ***N*** = **133**				**15 km**, ***N*** = **163**				**10 mi**, ***N*** = **155**				**20 km**, ***N*** = **113**			
	**Sex**	***N***	**Mean**	**Sd**	***p***	***N***	**Mean**	**Sd**	***p***	***N***	**Mean**	**Sd**	***p***	***N***	**Mean**	**Sd**	***p***
**AGE**																	
[3,20)	Men	8	00:43:04	00:11:18	0.641	13	01:02:57	00:22:13	0.862	13	01:01:38	00:17:54	0.850	7	01:07:25	00:17:10	0.117
[3,20)	Women	6	00:45:42	00:09:21		12	01:01:41	00:12:35		11	01:02:51	00:13:02		8	01:21:59	00:16:07	
[20,30)	Men	11	00:34:05	00:00:11	<0.001	10	00:41:47	00:00:21	<0.001	11	00:45:17	00:00:18	<0.001	10	00:56:36	00:00:35	<0.001
[20,30)	Women	10	00:38:36	00:00:33		10	00:47:40	00:00:46		10	00:51:59	00:00:29		10	01:04:50	00:00:54	
[30,40)	Men	10	00:34:24	00:00:26	<0.001	10	00:42:42	00:00:41	<0.001	9	00:46:10	00:00:48	<0.001	10	00:58:12	00:00:57	<0.001
[30,40)	Women	9	00:39:04	00:00:47		10	00:48:10	00:01:02		10	00:52:22	00:01:09		10	01:05:46	00:01:33	
[40,50)	Men	10	00:37:30	00:01:27	<0.001	10	00:45:44	00:01:09	<0.001	11	00:49:15	00:01:56	<0.001	10	01:05:13	00:04:07	0.002
[40,50)	Women	11	00:42:25	00:02:14		10	00:50:45	00:00:57		10	00:55:23	00:01:12		10	01:11:08	00:03:12	
[50,60)	Men	10	00:41:20	00:01:03	<0.001	10	00:51:04	00:02:00	<0.001	9	00:53:45	00:02:20	<0.001	10	01:11:28	00:04:34	0.001
[50,60)	Women	10	00:47:15	00:02:56		10	00:58:33	00:02:31		10	01:02:08	00:02:29		8	01:22:25	00:06:01	
[60,75)	Men	14	00:48:12	00:06:14	0.005	15	00:56:59	00:01:51	<0.001	16	01:01:08	00:04:52	<0.001	11	01:24:29	00:09:31	0.053
[60,75)	Women	13	00:57:08	00:08:33		15	01:10:36	00:08:07		14	01:12:13	00:05:04		6	01:40:03	00:14:56	
[75,100)	Men	9	01:15:56	00:15:19	0.096	15	01:36:28	00:42:00	0.105	13	01:24:07	00:16:26	0.028	2	01:32:03	00:02:40	
[75,100)	Women	2	01:06:10	00:01:39		13	01:59:05	00:28:35		8	01:40:48	00:14:44		1	01:58:51		
**COUNTRY**																	
USA	Men	39	00:51:45	00:16:38	0.469	23	01:24:09	00:40:17	0.690	26	01:15:30	00:17:25	0.512	17	01:18:48	00:10:20	0.183
USA	Women	33	00:49:22	00:10:48		37	01:20:07	00:33:09		27	01:12:11	00:19:06		23	01:24:57	00:18:04	
CAN	Men					13	01:02:10	00:06:06	0.063								
CAN	Women					2	00:58:35	00:00:42									
ETH-KEN	Men	18	00:34:14	00:00:21	<0.001	21	00:42:17	00:00:52	<0.001	21	00:45:46	00:01:18	<0.001	16	00:57:42	00:02:34	<0.001
ETH-KEN	Women	12	00:39:11	00:00:55		16	00:48:04	00:00:58		11	00:52:27	00:01:01		11	01:05:28	00:01:39	
GBR	Men									19	00:54:07	00:05:42	0.003				
GBR	Women									17	01:05:03	00:12:14					
JPN	Men																
JPN	Women																
Other	Men	15	00:38:23	00:03:49	<0.001	26	00:50:03	00:07:05	0.002	16	00:53:43	00:07:40	0.082	27	01:08:03	00:12:38	0.157
Other	Women	16	00:45:25	00:04:47		25	01:01:55	00:16:28		18	01:00:52	00:14:38		19	01:12:30	00:08:22	
		**Half marathon**, ***N*** = **170**				**25 km**, ***N*** = **139**				**30 km**, ***N*** = **119**				**Marathon**, ***N*** = **170**			
	**Sex**	***N***	**Mean**	**Sd**	***p***	***N***	**Mean**	**Sd**	***p***	***N***	**Mean**	**Sd**	***p***	***N***	**Mean**	**Sd**	***p***
**AGE**																	
[3,20)	Men	15	01:26:54	00:32:50	0.922	5	01:36:03	00:37:41	0.805	4	02:12:12	01:03:27	0.736	13	03:19:45	01:38:07	0.416
[3,20)	Women	15	01:27:56	00:23:50		8	01:40:42	00:16:40		3	02:27:41	00:51:08		14	02:55:30	00:37:18	
[20,30)	Men	10	00:58:46	00:00:15	<0.001	10	01:12:28	00:00:59	<0.001	11	01:29:16	00:01:04	<0.001	10	02:04:17	00:00:32	<0.001
[20,30)	Women	10	01:05:53	00:00:45		10	01:23:51	00:01:59		11	01:42:54	00:02:38		10	02:19:42	00:02:16	
[30,40)	Men	9	00:59:38	00:00:38	<0.001	10	01:15:29	00:01:28	<0.001	10	01:30:06	00:02:15	<0.001	10	02:04:31	00:01:22	<0.001
[30,40)	Women	10	01:07:05	00:01:18		10	01:25:26	00:01:52		10	01:44:34	00:02:32		10	02:19:54	00:02:12	
[40,50)	Men	11	01:04:16	00:02:05	<0.001	10	01:20:27	00:02:52	<0.001	10	01:39:54	00:08:05	<0.001	10	02:13:19	00:04:21	<0.001
[40,50)	Women	10	01:12:08	00:02:07		10	01:31:10	00:04:33		10	01:55:15	00:04:28		10	02:29:22	00:03:27	
[50,60)	Men	10	01:10:37	00:02:14	<0.001	10	01:29:11	00:03:35	<0.001	9	01:49:41	00:02:35	<0.001	10	02:27:17	00:05:28	<0.001
[50,60)	Women	10	01:20:42	00:03:35		10	01:45:49	00:06:59		8	02:05:26	00:06:34		10	02:49:44	00:09:26	
[60,75)	Men	15	01:19:44	00:06:01	<0.001	15	01:44:32	00:09:40	<0.001	13	02:11:43	00:19:33	0.007	15	02:49:11	00:08:27	<0.001
[60,75)	Women	15	01:35:06	00:07:16		15	02:12:39	00:14:19		11	02:38:56	00:23:40		15	03:24:49	00:15:00	
[75,100)	Men	17	02:02:09	00:32:27	0.240	12	02:27:40	00:27:13	0.138	7	03:12:03	00:31:43	0.177	16	04:19:40	01:10:47	0.010
[75,100)	Women	13	02:20:38	00:47:10		4	02:41:45	00:08:36		2	03:55:42	00:25:13		17	05:48:17	01:48:55	
**COUNTRY**																	
USA	Men	19	01:46:01	00:37:14	0.315	38	01:54:22	00:31:09	0.574	21	02:31:09	00:45:22	0.761	20	03:57:08	01:38:05	0.546
USA	Women	14	01:35:22	00:22:16		40	01:58:01	00:25:24		17	02:35:23	00:39:51		18	04:19:24	02:03:45	
CAN	Men	10	01:32:52	00:10:38	0.021									12	03:11:16	00:21:04	0.042
CAN	Women	9	02:26:20	00:56:01										5	06:09:41	02:15:30	
ETH-KEN	Men	22	01:00:02	00:01:42	<0.001	14	01:13:41	00:02:21	<0.001	10	01:29:33	00:02:52	<0.001	28	02:06:21	00:03:54	<0.001
ETH-KEN	Women	21	01:06:54	00:01:51		11	01:23:32	00:01:40		4	01:40:22	00:02:19		10	02:19:46	00:01:32	
GBR	Men	14	01:17:12	00:21:39	0.069												
GBR	Women	6	01:43:24	00:27:03													
JPN	Men									12	01:30:08	00:01:06	<0.001	3	02:36:22	00:01:55	0.014
JPN	Women									17	01:46:36	00:04:20		10	03:22:05	00:47:21	
Other	Men	22	01:20:24	00:30:42	0.665	20	01:20:55	00:06:32	<0.001	21	01:49:41	00:11:27	0.031	21	02:48:30	00:52:22	0.654
Other	Women	33	01:23:28	00:14:17		16	01:31:34	00:05:53		17	02:05:56	00:27:01		43	02:54:26	00:42:31	

*P-values of t-test of mean performances between sexes were shown*.

Regarding country, in all races differences were significant (*p* < 0.001) between sex performances for Kenya and Ethiopia. For Canada, significant differences were found only in 8 km (*p* = 0.009), half-marathon (*p* = 0.021), and marathon (*p* = 0.042). In Great Britain there were significant differences in 8 km (*p* = 0.011) and 10 mi (*p* = 0.003), in Japan in 30 km (*p* < 0.001) and marathon (*p* = 0.014). For other country groups, significant differences, between sex performances, were found in most of all races.

### Statistical model

In Table 2, for each race from 5 km to marathon, the estimates and confidence intervals of fixed effects have been reported. In all races, women were significantly slower than men (*p* < 0.001). The estimated sex differences ranged from a minimum of 0.003 (00:04:19 h:min:s), reached in 4 mi and 8 km, to a maximum of 0.019 (00:27:22 h:min:s) reached in marathon. Therefore, from 8 km onward, estimated sex differences were increasing.

**Table 2:** Non-linear regression analysis (mixed model) of world records (5 km to marathon).

	**5 km**	**4 mi**	**8 km**	**10 km**	**12 km**	**15 km**	**10 mi**	**20 km**	**Half-marathon**	**25 km**	**30 km**	**Marathon**
	***Estimate (CI)***	***Estimate (CI)***	***Estimate (CI)***	***Estimate (CI)***	***Estimate (CI)***	***Estimate (CI)***	***Estimate (CI)***	***Estimate (CI)***	***Estimate (CI)***	***Estimate (CI)***	***Estimate (CI)***	***Estimate (CI)***
**FIXED PARTS**												
(Intercept)	0.013 (0.011–0.014)^***^	0.017 (0.016–0.017)^***^	0.020 (0.020–0.021)^***^	0.026 (0.024–0.027) ^***^	0.030 (0.029–0.031)^***^	0.043 (0.040–0.046)^***^	0.041 (0.040–0.043)^***^	0.047 (0.045–0.049)^***^	0.056 (0.052–0.060) ^***^	0.065 (0.064–0.066)^***^	0.081 (0.078–0.085)^***^	0.122 (0.116–0.128)^***^
Sex:W	0.004 (0.002–0.005)^***^	0.003 (0.002–0.003)^***^	0.003 (0.002–0.004)^***^	0.004 (0.003–0.005) ^***^	0.004 (0.003–0.006)^***^	0.005 (0.002–0.008)^***^	0.006 (0.005–0.008)^***^	0.008 (0.006–0.010)^***^	0.007 (0.004–0.010)^***^	0.012 (0.011–0.013)^***^	0.014 (0.011–0.017)^***^	0.019 (0.014–0.024)^***^
**AGE**												
P_Age_	0.038 (0.024–0.051)^***^	0.039 (0.032–0.047)^***^	0.049 (0.043–0.055)^***^	0.065 (0.057–0.073)^***^	0.064 (0.056–0.073)^***^	0.124 (0.095–0.153)^***^	0.064 (0.053–0.074)^***^	0.053 (0.041–0.065)^***^	0.094 (0.071–0.117)^***^	0.131 (0.119–0.143)^***^	0.166 (0.139–0.193)^***^	0.272 (0.223–0.321)^***^
${\text{P}}_{\text{Age}}^{2}$	0.055 (0.043–0.068)^***^	0.056 (0.050–0.062)^***^	0.069 (0.063–0.074)^***^	0.058 (0.052–0.065)^***^	0.064 (0.057–0.071)^***^	0.126 (0.104–0.147)^***^	0.078 (0.070–0.086)^***^	0.049 (0.038–0.060)^***^	0.159 (0.138–0.179) ^***^	0.126 (0.117–0.136)^***^	0.162 (0.140–0.184)^***^	0.490 (0.446–0.534)^***^
${\text{P}}_{\text{Age}}^{3}$	0.005 (−0.007–0.016)	0.002 (−0.004–0.009)	0.020 (0.015–0.025)^***^	0.001 (−0.005–0.008)	0.005 (−0.003–0.012)	−0.008 (−0.032–0.015)	−0.016 (−0.024–−0.007)^***^	−0.032 (−0.043–−0.021)^***^	−0.036 (−0.054–−0.019) ^***^	−0.011 (−0.021–−0.002)^*^	−0.024 (−0.047–−0.002)^*^	−0.114 (−0.159–−0.069)^***^
${\text{P}}_{\text{Age}}^{4}$	0.031 (0.020–0.042)^***^	0.028 (0.023–0.034)^***^	0.040 (0.035–0.045)^***^	0.034 (0.027–0.040)^***^	0.020 (0.013–0.027)^***^	0.066 (0.042–0.089)^***^	0.045 (0.037–0.053)^***^	0.014 (0.004–0.023)^**^	0.087 (0.069–0.106)^***^	0.073 (0.065–0.082)^***^	0.100 (0.081–0.120)^***^	0.239 (0.201–0.277)^***^
**COUNTRY (REF. USA)**												
CAN	0.007 (0.003–0.010)^***^	−0.000 (−0.003–0.002)	0.000 (−0.001–0.001)	−0.002 (−0.005–0.000)		−0.006 (−0.016–0.004)			0.013 (0.004–0.022)^**^			−0.013 (−0.026–−0.000)^*^
ETH–KEN	0.001 (−0.001–0.003)	−0.000 (−0.001–0.001)	−0.000 (−0.001–0.001)	−0.000 (−0.002–0.001)	−0.001 (−0.003–0.001)	−0.000 (−0.005–0.005)	−0.001 (−0.004–0.001)	−0.001 (−0.004–0.002)	0.001 (−0.005–0.006)	0.000 (−0.002–0.003)	−0.003 (−0.009–0.003)	0.008 (−0.001–0.017)
GBR	0.000 (−0.002–0.003)	0.001 (−0.000–0.002)	−0.001 (−0.002–0.000)	−0.000 (−0.002–0.001)			−0.002 (−0.004–−0.000) ^*^		0.001 (−0.006–0.008)			
JPN											−0.002 (−0.007–0.004)	0.001 (−0.010–0.012)
Other	0.000 (−0.002–0.003)	−0.000 (−0.002–0.001)	−0.001 (−0.002–0.000)	−0.001 (−0.002–0.000)	−0.001 (−0.002–0.001)	−0.003 (−0.007–0.000)	−0.001 (−0.003–0.001)	−0.001 (−0.003–0.001)	−0.000 (−0.005–0.004)	−0.001 (−0.003–0.001)	−0.003 (−0.007–0.001)	−0.001 (−0.007–0.006)
**SEX** × **AGE**												
W × P_Age_	0.040 (0.022–0.058)^***^	0.017 (0.005–0.028)^**^	0.020 (0.011–0.028)^***^	0.033 (0.023–0.044)^***^	0.017 (−0.001–0.034)	0.041 (0.003–0.078) ^*^	0.040 (0.026–0.055)^***^	0.022 (0.004–0.039) ^*^	0.066 (0.030–0.102)^***^	0.078 (0.063–0.094)^***^	0.066 (0.033–0.099)^***^	0.291 (0.231–0.350)^***^
W × ${\text{P}}_{\text{Age}}^{2}$	0.032 (0.014–0.049)^***^	0.001 (−0.012–0.013)	−0.007 (−0.015–0.002)	0.029 (0.019–0.039)^***^	0.013 (−0.008–0.035)	0.036 (0.005–0.068)^*^	0.027 (0.013–0.041) ^***^	0.031 (0.013–0.049)^**^	0.029 (−0.001–0.059)	0.016 (−0.002–0.033)	0.052 (0.018–0.086)^**^	0.031 (−0.026–0.087)
W × ${\text{P}}_{\text{Age}}^{3}$	0.018 (0.002–0.034) ^*^	0.004 (−0.008–0.016)	−0.008 (−0.016–0.001)	0.020 (0.011–0.030)^***^	0.002 (−0.019–0.023)	0.032 (0.002–0.062)^*^	0.015 (0.002–0.027)^*^	0.006 (−0.013–0.024)	0.034 (0.009–0.059)^**^	0.008 (−0.009–0.026)	0.023 (−0.010–0.056)	0.221 (0.166–0.277)^***^
W × ${\text{P}}_{\text{Age}}^{4}$	0.002 (−0.012–0.017)	−0.014 (−0.025–−0.004)^**^	−0.024 (−0.033–0.016)^***^	−0.004 (−0.013–0.005)	0.004 (−0.014–0.023)	−0.013 (−0.047–0.022)	−0.024 (−0.037–−0.011)^***^	−0.002 (−0.019–0.015)	−0.005 (−0.030–0.020)	−0.040 (−0.056–−0.023)^***^	−0.023 (−0.055–0.009)	−0.011 (−0.068–0.045)
**RANDOM PARTS**												
N_runners_	105	98	121	105	112	114	99	91	109	98	89	109
ICC	0.918	0.869	0.786	0.846	0.859	0.821	0.903	0.807	0.961	0.135	0.593	0.557
*N*	183	151	167	171	133	163	155	113	170	139	119	170
*R*^2^/$\Omega_{0}^{2}$	0.993/0.993	0.995/0.995	0.996/0.996	0.996/0.996	0.997/0.997	0.989/0.989	0.997/0.997	0.991/0.991	0.998/0.998	0.981/0.981	0.985/0.984	0.988/0.988

Estimates and confidence intervals (CI) of fixed effects were shown as numeric values. To convert them in h:min:s, one should multiply estimates by 1,440 (minutes per day). The integers obtained are minutes and the fraction should be multiplied by 60 to obtain the seconds. Effects of sex were reported with men being the reference gender group and effects on countries were reported with USA being the reference group. P-values ranges were remarked using ^^*^^ (see note). For the random effects, the number of clusters (N_runners_), ICC (intra-cluster correlation), the number of observations (N), the r-squared approximation (R^2^) together with a simplified version of the Omega-squared value ($\Omega_{0}^{2}$) were reported.

* p < 0.05

** p < 0.01

*** *p < 0.001*.

Regarding country, there were no significant differences between nationalities except in these cases: (1) runners from Canada were significantly slower than runners from the United States of America in 5 km (estimated difference 0.007, 00:10:05 h:min:s, *p* < 0.001) and half-marathon (estimated difference 0.013, 00:18:43 h:min:s, *p* < 0.01) but significantly faster in marathon (estimated difference −0.013, 00:18:43 h:min:s, *p* < 0.05); (2) runners from Great Britain were significantly faster than runners from the United States of America in 10 miles (estimated difference −0.002, 00:02:53 h:min:s, *p* < 0.05).

From Table 2, the values of *R*^2^ were high for all races. This meant a good fit, for each model. Intra-class correlations (ICC) were also high, except in 25 km where ICC was poor. In 30 km and marathon ICC were fair.

**Figure 1:**
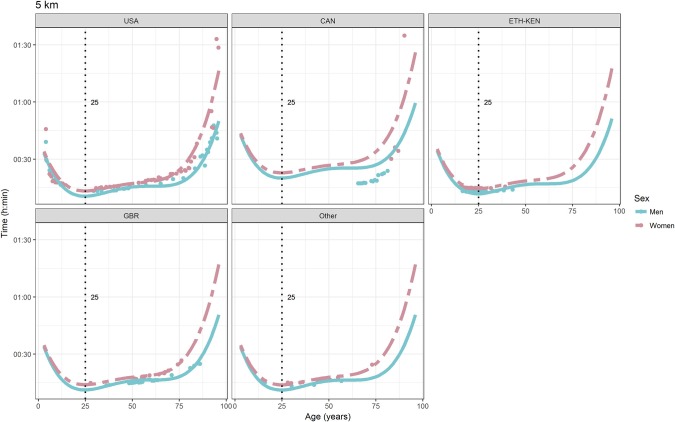
World record in 5 km by sex, age (in years), and country. Points were observations. Curves represented quartic degree polynomial regression. Vertical line with the numeric label was the age at peak performance.

**Figure 2:**
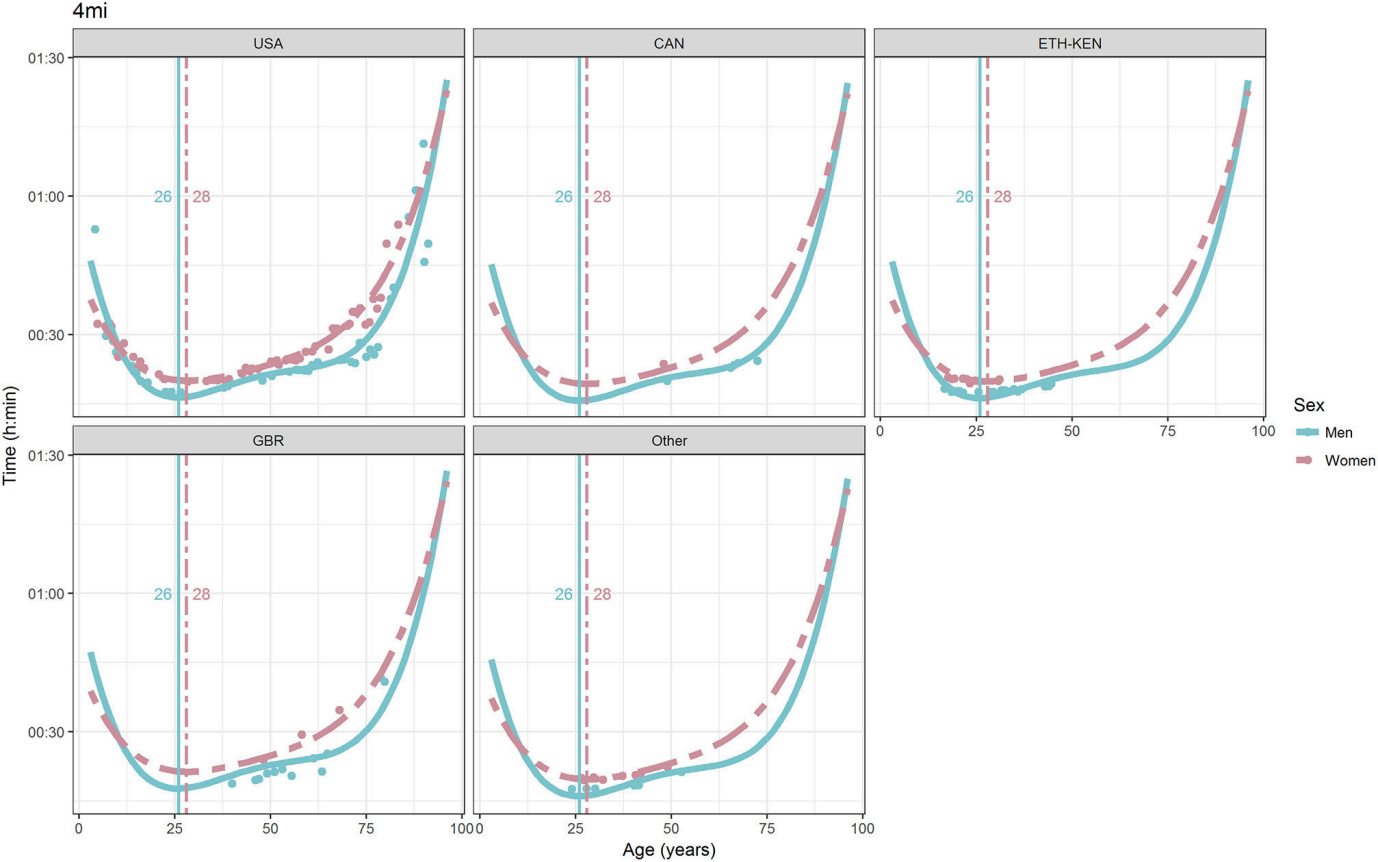
World record in 4 miles by sex, age (in years), and country. Points were observations. Curves represented quartic degree polynomial regression. Vertical lines with numeric labels were the ages at peak performance.

**Figure 3:**
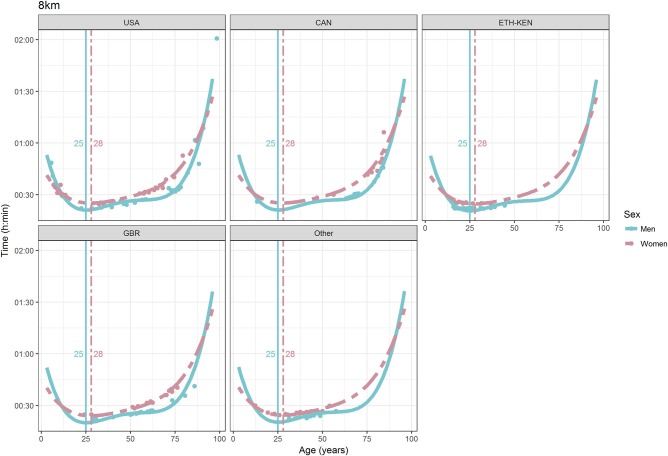
World record in 8 km by sex, age (in years), and country. Points were observations. Curves represented quartic degree polynomial regression. Vertical lines with numeric labels were the ages at peak performance.

**Figure 4:**
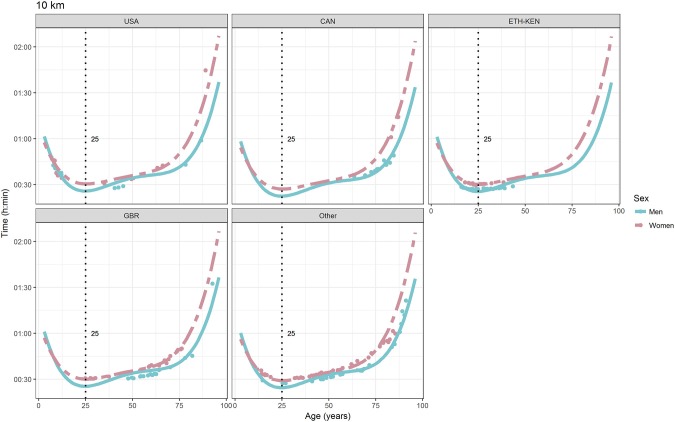
World record in 10 km by sex, age (in years), and country. Points were observations. Curves represented quartic degree polynomial regression. Vertical line with numeric label was the age at peak performance.

**Figure 5:**
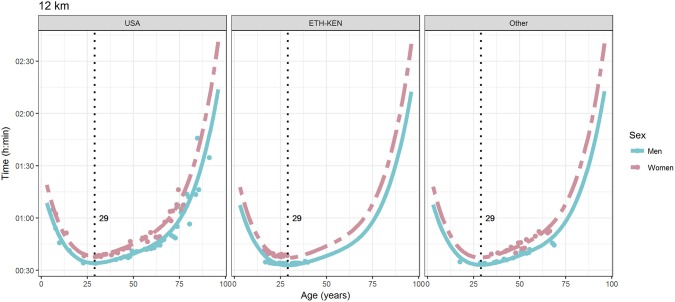
World record in 12 km by sex, age (in years), and country. Points were observations. Curves represented quartic degree polynomial regression. Vertical line with numeric label was the age at peak performance.

**Figure 6:**
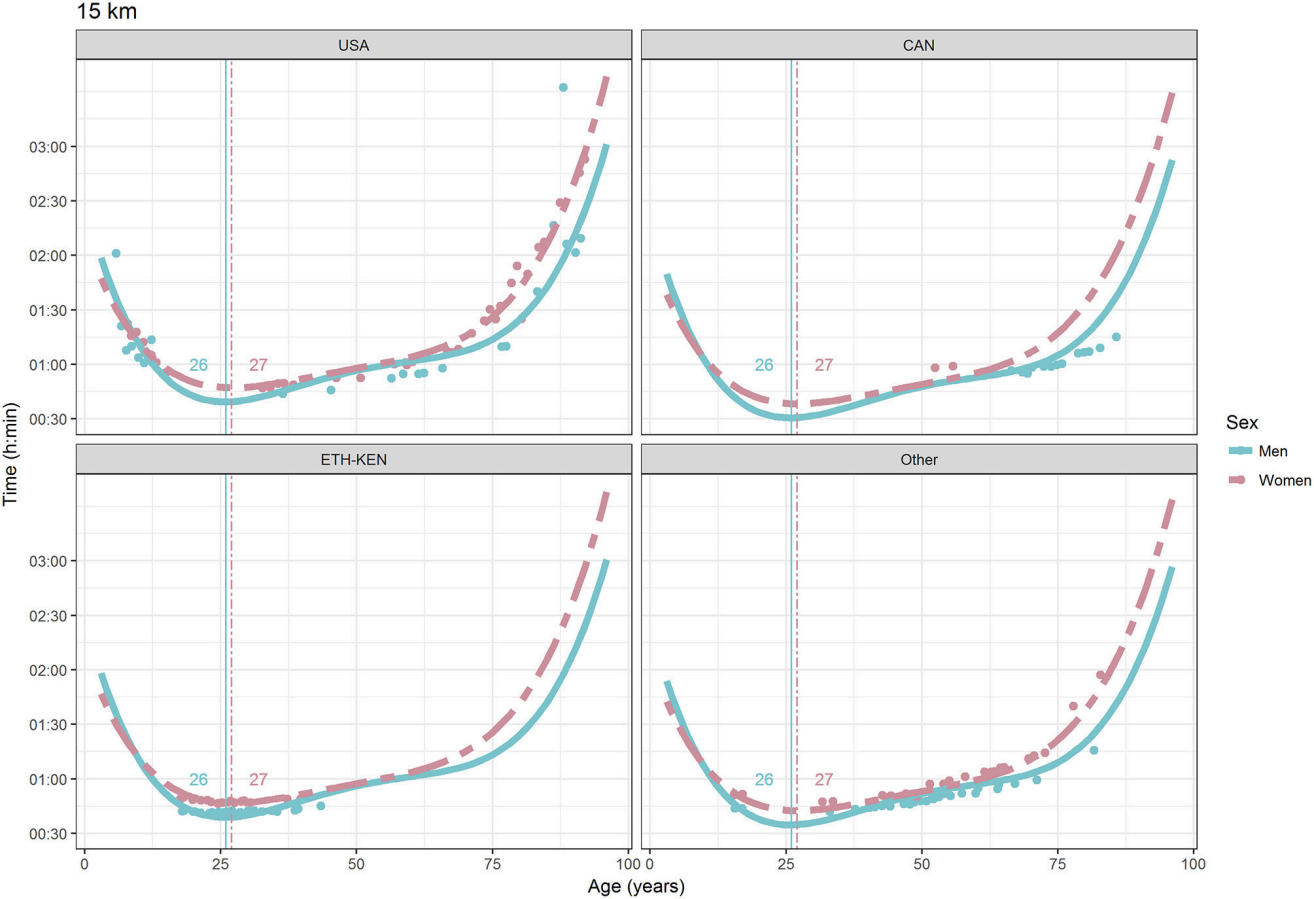
World record in 15 km by sex, age (in years), and country. Points were observations. Curves represented quartic degree polynomial regression. Vertical lines with numeric labels were the ages at peak performance.

**Figure 7:**
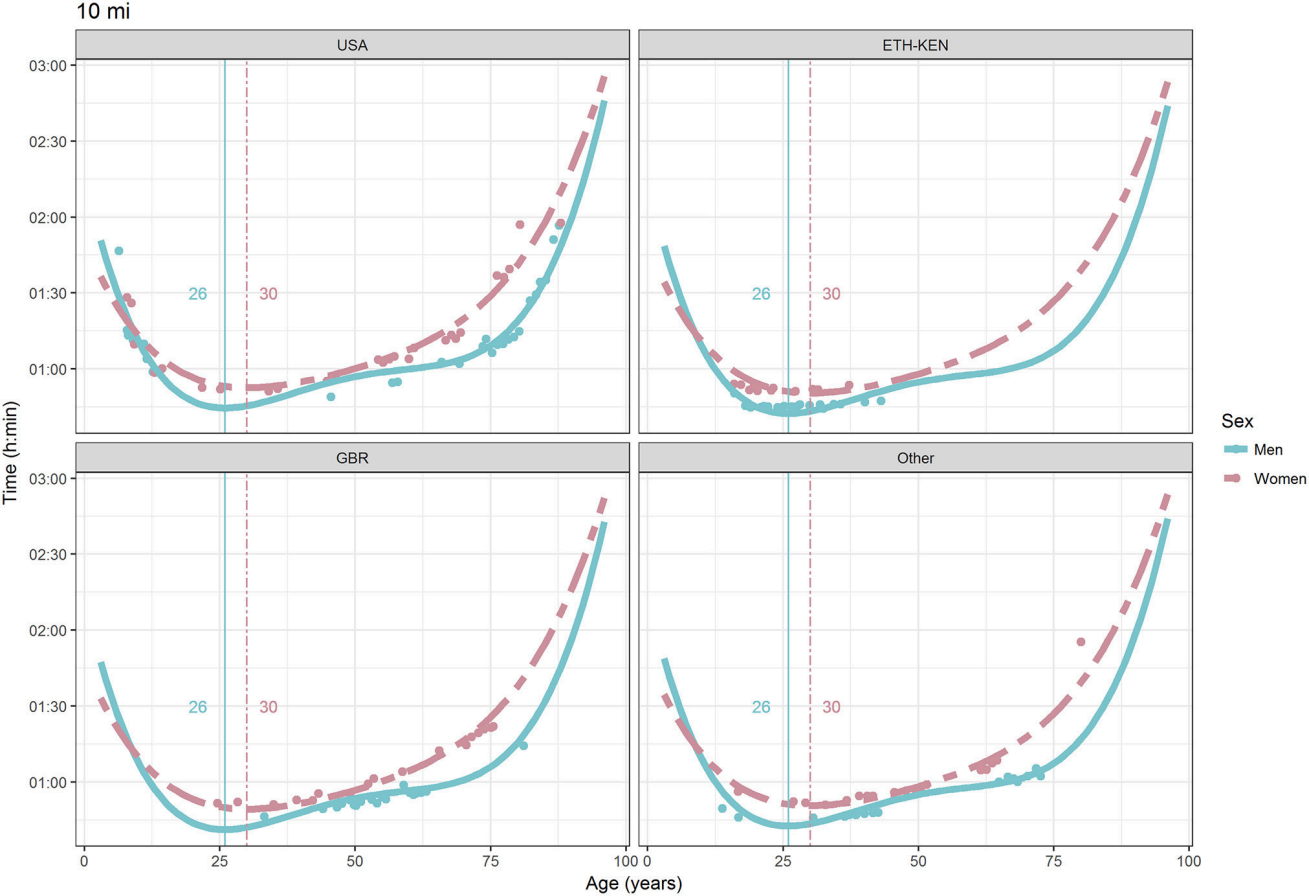
World record in 10 miles by sex, age (in years), and country. Points were observations. Curves represented quartic degree polynomial regression. Vertical lines with numeric labels were the ages at peak performance.

**Figure 8:**
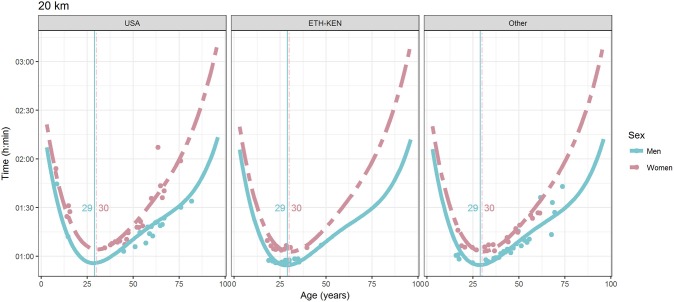
World record in 20 km by sex, age (in years), and country. Points were observations. Curves represented quartic degree polynomial regression. Vertical lines with numeric labels were the ages at peak performance.

**Figure 9:**
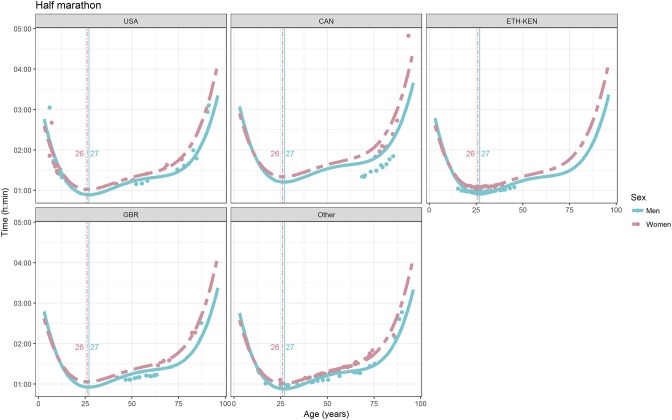
World record in half-marathon by sex, age (in years), and country. Points were observations. Curves represented quartic degree polynomial regression. Vertical lines with numeric labels were the ages at peak performance.

**Figure 10:**
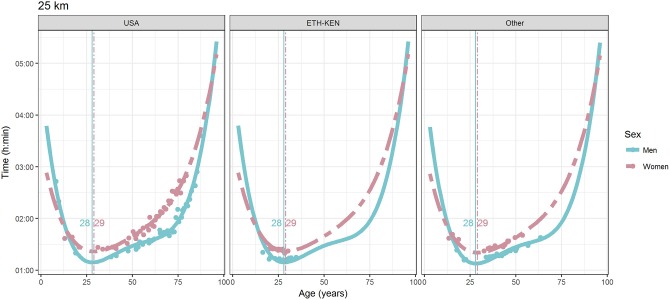
World record in 25 km by sex, age (in years), and country. Points were observations. Curves represented quartic degree polynomial regression. Vertical lines with numeric labels were the ages at peak performance.

**Figure 11:**
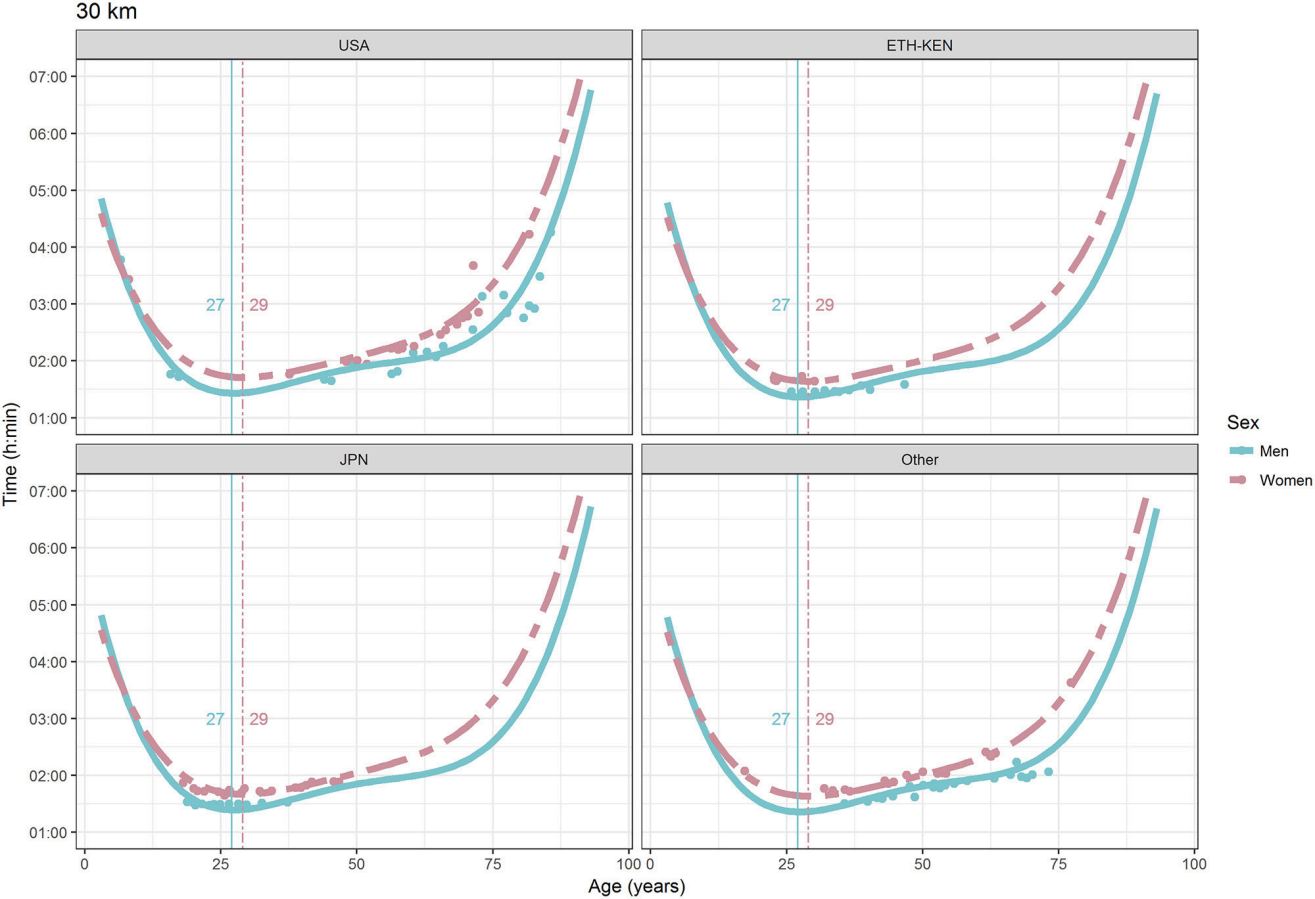
World record in 30 km by sex, age (in years), and country. Points were observations. Curves represented quartic degree polynomial regression. Vertical lines with numeric labels were the ages at peak performance.

**Figure 12:**
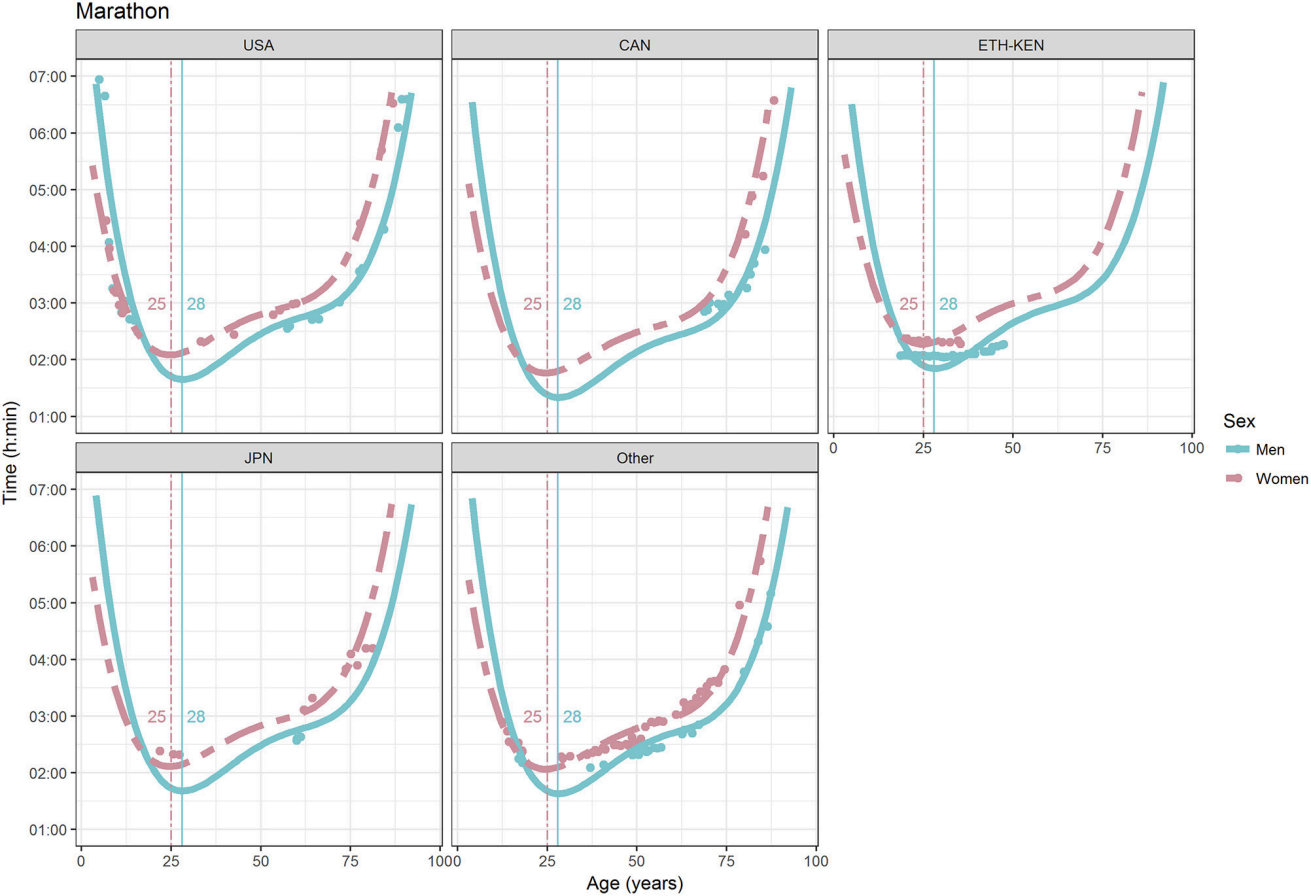
World record in marathon by sex, age (in years), and country. Points were observations. Curves represented quartic degree polynomial regression. Vertical lines with numeric labels were the ages at peak performance.

For each race, all the effects in Table 2, together with the age of peak performances, were shown graphically in Figures 1–12. It could be observed that performances improved, reaching a minimum fastest time, and then they worsened with age. For each race, men performances, in the first part of the curve, were not always better than women performances, in particular in 25 km and in marathon, but men race times improved faster compared with women. These effects have been explained by the coefficients Sex × Age reported in Table 2.

As shown in Figures 9, 12, the age of peak performance in half-marathon and marathon for men was, respectively, 27 and 28 years. Instead, women achieved their best half-marathon and marathon race time earlier in life than men. The difference was, respectively, of 1 and 3 years. On the contrary, in the other races, the best women performances were achieved later in life than men or at the same. The differences were: 2 years later in 4 miles and 30 km (Figures 2, 11), 3 years later in 8 km (Figure 3), 1 year later in 15–20–25 km (Figures 6, 8, 10), and 4 years later in 10 miles (Figure 7). In 5, 10, and 12 km (Figures 1, 4, 5) the age of peak performance was equal for both sexes: 25 years for the first two races and 29 years for the latter. Therefore, there was, apparently, no relationship in the age of peak performances with increasing race distance.

**Figure 13:**
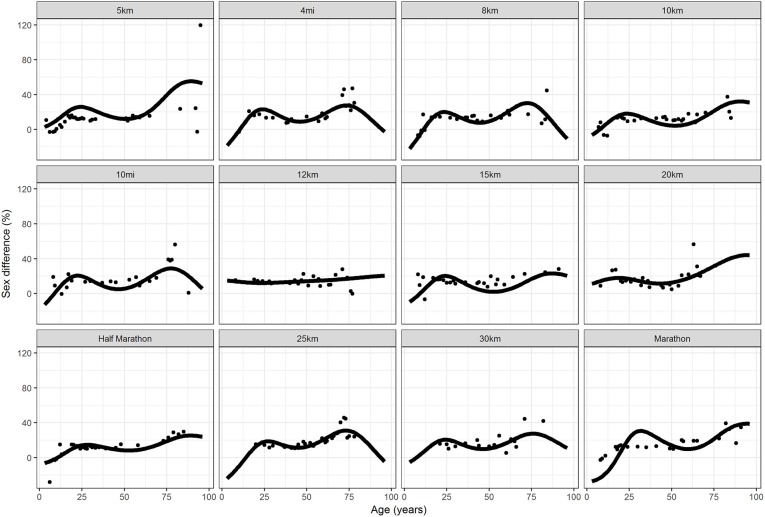
Sex differences by age (in years) from 5 km to marathon. Points were observations. Curves represented fitted values, with country being the reference group (USA).

In Figure 13, the sex differences by ages and by race, from 5 km to marathon were shown. The fitted values lines were calculated from the model in Table 2 with country factor at the reference group level (USA). Except in 12 km, where sex differences appeared quite constant on age, even if a little higher in the older ages, in all other races sex difference had two maximum: they increased in the younger ages, until an age near the age of peak performance, then, after decreasing, they increased again reaching an absolute maximum in the older ages.

**Figure 14:**
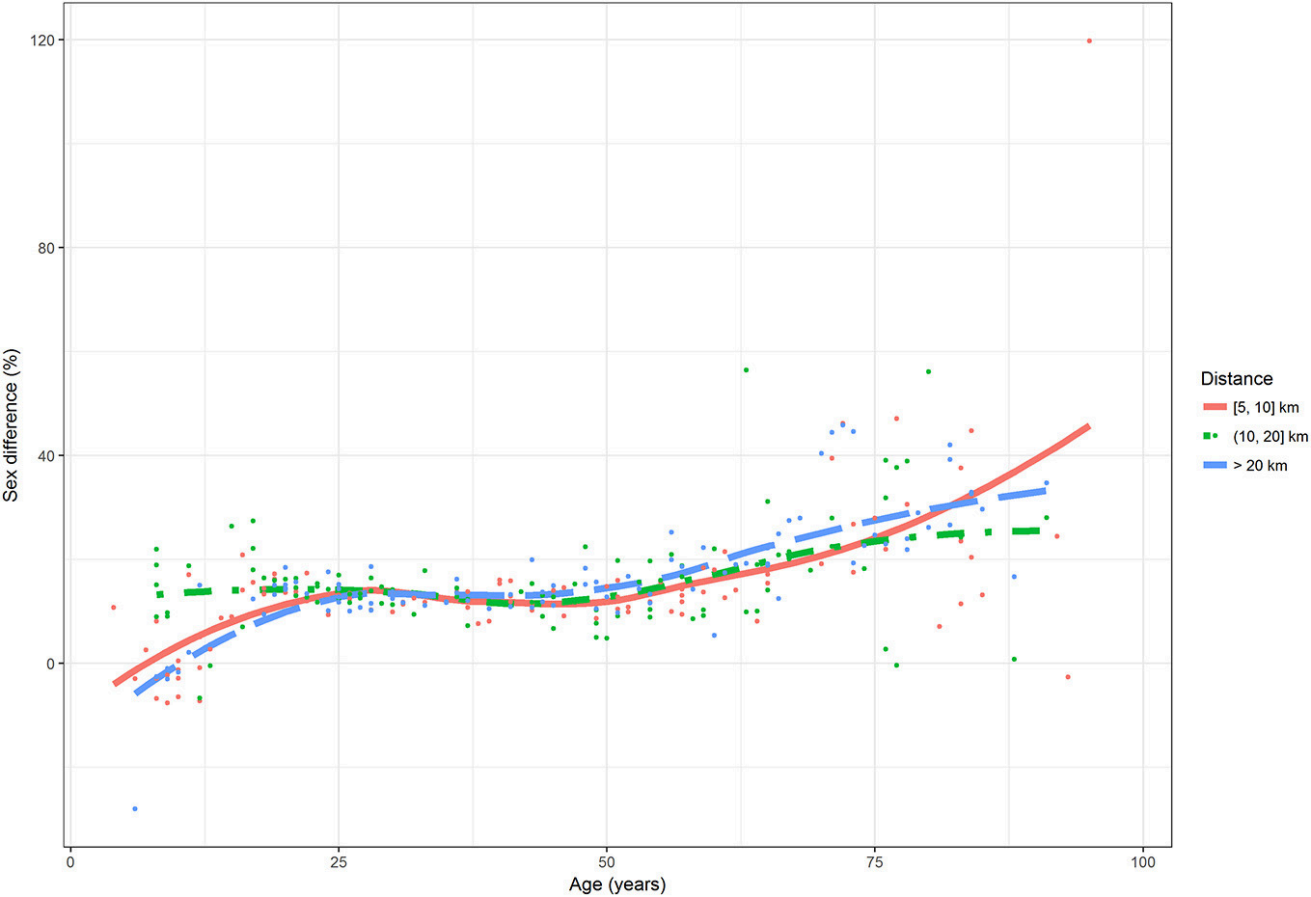
Sex differences by age (in years) and by distance. Points were observations. Curves represented smoothed values.

The relationship between sex differences with age and by distance in km was shown in Figure 14. Points were the observed differences, for all countries and races, and lines were the smoothed trends at the three classes of distance, [5, 10], (10, 20], and >20 km. It could be observed that the trend of sex differences in shortest distances, from 5 to 10 km included is more similar to the trend of sex differences in longest distances, >20 km, except for the older ages, where the sex differences were more increasing in shortest distances. In younger ages, the sex differences were highest in distances from 10 to 20 km and lowest in longest distances. In older ages, on the contrary, the sex differences were lowest in distances from 10 to 20 km and highest in shortest distances.

## Discussion

The aim of this study was to analyze the world single age records from 5 km to marathon with the hypothesis to find a lower age of peak running performance in the shorter compared to the longer running distances. The most important findings were (i) differences in the age of peak performance between women and men (e.g., women achieved their best half-marathon and marathon race time earlier in life than men), and (ii) for nearly all distances sex differences showed an absolute maximum at old ages and relative maximum near the age of peak performance. The disagreement between our research hypothesis and the main finding concerning the lack of variation of the age of peak performance by distance might be due to the different methodological approaches adopted in the present study compared with previous research which was conducted either in ultra-endurance running (Knechtle et al., 2014; Nikolaidis and Knechtle, 2018a,b) or other exercise modes (Baker and Tang, 2010; Berthelot et al., 2012; Elmenshawy et al., 2015). This study was conducted on world records, i.e., the best runners were selected, in contrast with previous research that used all runners to model the age of peak performance. When all runners were included in this model, the variation of participation by age might influence this model. For instance, a larger participation in a specific age would be likely associated with slower performance and *vice versa*.

### Differences in performance between countries

In this study, it was found that (i) runners from Canada were slower than runners from the United States of America, in 5 km and half-marathon, but faster in marathon; and (ii) runners from Great Britain were significantly faster than runners from the United States of America in 10 miles. Nonetheless, the reason for these cases should be due to the different age distribution within countries. In fact, in case (i) above, for Canada, there were only observations at the age of 65 years and older. In marathon, instead, there were, for USA, few observations in the central age classes and more or less the same number of observations in the older ages. In 10 miles, in USA, compared to GBR, the number of observations in the older ages was greater.

### Differences in the age of peak performance between women and men

A first important finding was that women achieved their best half-marathon and marathon race time earlier in life than men. Half-marathon and marathon are very popular race distances and the actual findings confirm findings from earlier studies where women achieved their best race times earlier in life than men in both half-marathon (Knechtle and Nikolaidis, 2018; Nikolaidis et al., 2018) and marathon (Nikolaidis et al., 2018) running. The age of peak performance was in agreement with the range (25–34 years old) proposed by Zavorsky et al. (2017). Furthermore, the age of peak performance in the present study was younger than what was reported in marathon (~34 years old) in a study that analyzed all finishers (Lehto, 2016).

Overall, the age of peak performance seems to depend from the duration and/or distance of the performance with a clear trend to increase with increasing duration and/or distance (Allen and Hopkins, 2015). Regarding the age of peak running performance, longer distances than the marathon distance (e.g., 50 and 100-km ultra-marathon) have been investigated where women seemed to achieve their best race time in 50-km ultra-marathon later in life compared to men (Nikolaidis and Knechtle, 2018a). In contrast, the age of peak performance was younger in women than in men in 100-km ultra-marathon running (Nikolaidis and Knechtle, 2018b).

Obviously, the age of peak running performance seems not to follow a specific pattern regarding the actual findings (e.g., the best women performances were achieved later in life than men (i.e., 4 miles, 15 km, 10 miles, 20, 25, 30 km) or at the same age (i.e., 5, 10, 12 km), where the age of peak performance did not change monotonically with the distance of race). This aspect seems to be confirmed with the two ultra-marathon distances of 50 and 100 km.

To interpret the differences of age of peak performance in endurance running between women and men, the correlates of sex difference in performance should be considered. Since these correlates vary by age, it would be reasonable to assume that this variation would partly explain the corresponding variation of peak performance by age. With regards to physiological correlates, it has been supported that the main physiological factor explaining the 10–12% slower race times in women than in men at the elite level is maximal oxygen uptake (Joyner, 2017). Other physiological correlated include longer limb levers, greater muscle mass and lower fat mass in men than in women (Millard-Stafford et al., 2018). On the other hand, it has been proposed that the greater sex difference in race speed in marathon with age might be due to the lower number of women finishers than men, as the lower participation levels of women and less depth among women would amplify the physiological sex differences (Hunter and Stevens, 2013). Moreover, these physiological and participation factors vary by age. For instance, the maximal oxygen uptake declines with age due to a reduction of muscle oxygen delivery (lower cardiac output) and of skeletal muscle oxidative capacity (mitochondrial dysfunction; Betik and Hepple, 2008). The decline of maximal oxygen uptake might be attenuated by endurance training (Rogers et al., 1990; Katzel et al., 2001). In addition, the participation rates in endurance running and the men-to-women ratio might also vary by age (Leyk et al., 2007; Lehto, 2016).

### Sex difference in performance

A second important finding was that sex differences showed an absolute maximum at old ages and relative maximum near the age of peak performance for nearly all distances. In other words, men are relatively faster at higher ages compared to women. This confirms earlier findings from marathon runners. When running times of the first 10 placed men and women in the 5-years age brackets between 20 and 79 years and the number of men and women who finished the “New York City Marathon” were analyzed for a 31-year period (1980–2010), the sex difference increased with advanced age and decreased across the 31 years, but more for the older age groups. The authors assumed that the greater sex difference in running speed that occurs with age was primarily explained by the lower number of women finishers than men (Hunter and Stevens, 2013). However, in the present study, only the performance of one woman to one man, in the specific 1-year age intervals, was compared.

The observation that the sex difference in performance increases with increasing age is most likely due to a specific discipline as the sex difference increases with age more for running than for swimming (Senefeld et al., 2016). For instance, this finding is in agreement with studies on endurance running such as half-marathon (Leyk et al., 2007) and marathon (Leyk et al., 2009; Hunter and Stevens, 2013). On the other hand, when compared with swimming, women were not slower compared to men in age groups 80–84 to 85–89 years when trends in participation, performance, and sex difference in performance of 65,584 freestyle master swimmers from 25–29 to 85–89 years competing in FINA World Masters Championships between 1986 and 2014 were investigated (Knechtle et al., 2016a). The same trends could also be observed for open-water freestyle swimming (Knechtle et al., 2017a) and other pool swimming disciplines such as breaststroke (Knechtle et al., 2016b), butterfly (Knechtle et al., 2017b), backstroke (Unterweger et al., 2016), and individual medley (Nikolaidis and Knechtle, 2018c). An explanation of this variation by exercise mode (i.e., running vs. swimming) might be that the physiological sex differences (for example, more subcutaneous fat in women) that limit women's performance more in weight-bearing exercise than non-weight bearing exercise, and more balanced participation levels of both sexes in elite swimming than marathon running (Senefeld et al., 2016; Millard-Stafford et al., 2018).

## Limitations

The first limitation is related to the design of the study. Data regarding single age of world records were retrieved from the website of a non-official sport organism (ARRS). Since they are all road races, caution needs to generalize these findings to events performed in stadium (e.g., 5 or 10 km). Second, the effect of nationality, a possible confounder of age, was quantified in the intercept of the statistical model. This improved the fit of the model and highlighted some differences between countries. Nevertheless, for each race there were not enough observations, by country, age, and sex, to better dealing with confounding and to investigate a significant sex difference in performance by country. For this reason, it was not possible to consider a three-way interaction statistical model sex × age × country or a simpler two-way interactions sex × country and age × country. The summary statistics provided in Table 1, could only give an idea about the sex difference effect by country. Nonetheless, the significance of *t*-test statistics is limited in its nature, and is not an accurate and reliable way to make inference such as a statistical model.

## Conclusions

This is the first study to investigate the age of peak performance, the sex difference in performance and the role of nationality in running distances from 5 km to marathon and it was found that differences seem to exist in the age of peak performance between women and men (e.g., women achieved their best half-marathon and marathon race time earlier in life than men), and for nearly all distances sex differences showed an absolute maximum at old ages and relative maximum near the age of peak performance. Considering the increased number of finishers in endurance running races during the last decades, the findings of the present study would have great practical interest for strength and conditioning coaches working with runners practicing endurance training regularly. Typically, a strength and conditioning coach trains runners in small groups including likely both women and men of various ages participating in endurance races that might vary for distance. Our findings highlighted the need for sex-specific training programs, especially near the age of peak performance and for elder runners. In addition, the lack of difference of the age of peak performance among race distances ranging from 5 km to marathon implied that strength and conditioning coaches of runners should set long-term performance goals considering the age of peak performance similarly for this range of distance.

## Author contributions

BK provided the data from the data base. SD performed the analysis. BK and PN drafted the manuscript. All authors approved the final version of the manuscript for publication.

### Conflict of interest statement

The authors declare that the research was conducted in the absence of any commercial or financial relationships that could be construed as a potential conflict of interest. The reviewer SF and handling editor declared their shared affiliation at the time of the review.
